# The association of urinary prostaglandins with uric acid in hyperuricemia patients

**DOI:** 10.1186/s12882-022-02928-y

**Published:** 2022-09-03

**Authors:** Huagang Lin, Ying Xu, Yuqi Zheng, Deping Wu, Zhibin Ye, Jing Xiao

**Affiliations:** 1 Department of Nephrology, Huadong Hospital Affiliated to Fudan University, Shanghai, 200040 P.R. China; 2 Shanghai Key Laboratory of Clinical Geriatric Medicine, Huadong Hospital Affiliated to Fudan University, Shanghai, P.R. China

**Keywords:** Hyperuricemia, Uric acid, Urinary prostaglandin, PGE2, Kidney

## Abstract

**Purpose:**

To explore the association between uric acid and urinary prostaglandins in male patients with hyperuricemia.

**Methods:**

A total of 38 male patients with hyperuricemia in outpatients of Huadong Hospital from July 2018 to January 2020 were recruited. Serum uric acid (SUA), 24 h urinary uric acid excretion and other indicators were detected respectively. 10 ml urine was taken to determine prostaglandin prostaglandin D (PGD), prostaglandin E1 (PGE1), prostaglandin E2 (PGE2), 6-keto-PGF1α, thromboxane A2 (TXA2) and thromboxane B2 (TXB2). Fraction of uric acid excretion (FEua) and uric acid clearance rate (Cua) were calculated. According to the mean value of FEua and Cua, patients were divided into two groups, respectively. The independent-samples *t* test and the Mann–Whitney U test were applied for normally and non-normally distributed data, respectively.

**Results:**

After adjusting confounding factors (age, BMI, eGFR, TG, TC, HDL and LDL), SUA was negatively correlated with urinary PGE1(*r* = -0.615, *P* = 0.009) and PGE2(*r* = -0.824, *P* < 0.001). Compared with SUA1 group (SUA < 482.6 mg/dl), SUA2 (SUA $\ge$ 482.6 mg/dl) had lower urinary PGE1(*P* = 0.022) and PGE2(*P* = 0.019) levels. Cua was positively correlated with PGE2 (*r* = 0.436, *P* = 0.01). The correlation persisted after adjustment for age, BMI, eGFR, TG, TC, HDL and LDL by multiple linear regression analysis. In the Cua1 group (Cua < 4.869 mL /min/1.73 m^2^), PGE2 were lower than that in Cua2 (Cua $\ge$ 4.869 mL /min/1.73 m^2^) group (*P* = 0.011).

**Conclusions:**

In male patients with hyperuricemia, SUA was negatively correlated with urinary PGE2, Cua was positively correlated with urinary PGE2. Urinary PGE2 were significantly different between different SUA and Cua groups.

## Introduction

Uric acid (UA) is the final product of purine metabolism [1]. High UA levels are known to be a risk factor for diabetes, Chronic kidney disease (CKD), and various cardiovascular diseases [2]. According to the data from the China National Health Survey (CNHS),the prevalence of hyperuricemia(HUA) was as high as 25.1% in men and 15.9% in women in mainland China [3], becoming a major public health problem and threatening public health. In recent decades, uric acid has attracted extensive interest because of the various peculiarities. Studies over the past two decades show that uric acid play a part in the oxidative stress, endothelial dysfunction and inflammation response [4–6]. Existing researches have suggested that uric acid is a risk factor for the development of chronic kidney disease by damaging renal tubule cells and affecting endothelial function and thus renal hemodynamics, however, the mechanism of how uric acid may affect renal hemodynamics is currently unknown.

Prostaglandins (PGs) are important lipid mediators produced from arachidonic acid via the sequential catalyzation of cyclooxygenases and specific prostaglandin synthases and PGs exert distinct roles by combining to a diverse family of membrane-spanning G protein-coupled prostanoid receptors [7]. As a kind of indispensable lipid mediator in human body, PGs are important in numerous physiological and pathophysiological processes. In the previous studies, considerable attention has been paid to inflammatory responses of prostaglandins. Under physiological situation, PGs play an important role in the regulation of renal hemodynamics, renin release, as well as water and salt balance [8–10]. Some in vitro studies have shown that uric acid may affect the release of cellular prostaglandins [11]. But the results have not been confirmed in patients.

Therefore, we made a reasonable hypothesis that renal local uric acid handling may be correlated with prostaglandins levels in hyperuricemia patients. Hence, the research was designed to study the association of uric acid with prostaglandins.

## Materials and methods

### Study participants

The patients who came to the outpatient of Nephrology in Huadong Hospital affiliated to Fudan University from July 2018 to January 2020 were screened and 38 male participants were recruited eventually. Each participant was in keeping with the following three conditions: first of all, the level of serum uric acid should be above the upper end of the normal range, which is 420 µmol/L (7 mg/dL) [12]; secondly, the health condition is generally well without any other chronic diseases or even obesity except hyperuricemia mentioned before; thirdly, there is no medication history within two weeks. In addition, to avoid dietary effects on uric acid metabolism, the patient who has greater use of high-fructose corn syrup as sweetener or drinking alcohol in the last three days will be excepted.

### Clinical and laboratory measurements

Each participant was guided to collect the 24-h urinary sample in a well-designed manner using a clean plastic bucket with lid. They were allowed to complete the process at home following the prescribed steps, recording all the time-points of specimens’ retention and filling necessary information in a standard questionnaire. It should be noted that 24-h urine collection must be done by discarding the first morning void and collecting all urine output for the next 24 h, including the first morning void the next day. During collection, the urine sample was required to store in an environment of 2–8℃ or in a cool and ventilated place and it should be well-blended after each urine retention.

On the morning of completing sample collection, each sample was promptly delivered to clinical laboratory for examination of 24-h urinary levels of uric acid, creatinine, sodium, potassium, glucose, albumin and urinary volume. The renal function and ability of handling of uric acid were estimated by relevant indicators, including clearance of creatinine (Ccr), clearance of uric acid (Cua), fractional excretion of uric acid (FEua). Clearance of creatinine was calculated from the formula Ccr = Uv × Ucr/Scr, expressed in ml/min (where Uv is urine volume/time, Ucr is urinary creatinine, and Scr is serum creatinine). Clearance of uric acid was calculated from the formula Cua = Uv × Uua/SUA, expressed in ml/min (where Uua is urinary uric acid and Sua is serum uric acid). Fractional excretion of uric acid was calculated as FEua = (Uua × Scr)/ (Sua × Ucr) × 100, expressed as percentage.

Another 10 ml of 24-h urine sample was centrifuged at 1500 rpm for 10 min, and 1.5 ml of supernatant was extracted with caution. The treated sample was frozen at − 80 °C until analysis. After harvesting them, we measured the concentrations of prostaglandins including PGD, PGE1, PGE2, PGI2 and TXA2. As we know, TXA2 primarily plays its role in the surrounding tissue by autocrine or paracrine manner. It is highly unstable in aqueous solution, where it spontaneously hydrolyses to the biologically inactive metabolite hemiacetal thromboxane B_2_ (TXA2-M) [13]. So does PGI2, its stable metabolite is 6-keto-prostaglandin F1α (PGI2-M) [14]. As a matter of course, PGI2 and TXA2 were assessed by the surrogate PGI2-M and TXA2-M. Respectively, commercially available enzyme-linked immunosorbent assays, with Human PGD ELISA kit, Human PGE1 ELISA kit, Human PGE2 ELISA kit, Human TXB2 ELISA kit and Human 6-k-PGF1α ELISA kit (all from Shanghai Jiwei Biological Technology Co., Ltd), were performed according to manufacturer’s instructions. These obtained levels of markers were normalized to urinary creatinine concentrations, and the results expressed as pg/ml.

After 12 h of fasting at night, venous blood was drawn from each participant for routine biochemical examination by skilled nurse. The representative indicators include serum levels of uric acid (SUA), creatinine (Scr), urinary nitrogen (BUN), total cholesterol (TC), triglycerides (TG), high-density lipoprotein (HDL), low-density lipoprotein (LDL) and fasting blood glucose (FBG). The estimated glomerular filtration rate (eGFR) (milliliters per minute per 1.73 m^2^) was calculated by the Chronic Kidney Disease Epidemiology Collaboration (CKD-EPI) formula [15].

Height and weight were measured by well-trained nurses in a standard process. Body mass index (BMI) was calculated as weight in kilograms divided by height in meters squared (kg/m^2^).

### Statistical analysis

The continuous variables are expressed as mean ± SD and categorical variables are reported in percentages. The normality of data was test by Shapiro Wilk (S-W test). The independent-samples *t* test and the Mann–Whitney U test were applied for normally and non-normally distributed data, respectively. Correlations were detected by Pearson’s or Spearman’s depending on the distribution of the data. If Pearson’s correlation analysis was statistically significant, multiple linear regression analysis was performed. Multiple linear regression analyses were performed to determine the association of urinary uric acid excretion with prostaglandins. Statistical significance for all analysis was set at *P* < 0.05. Statistical analysis was performed with software SPSS 22.0 and Prism 7.0a.

## Results

### The correlation between SUA and urinary prostaglandins

**Fig. 1:**
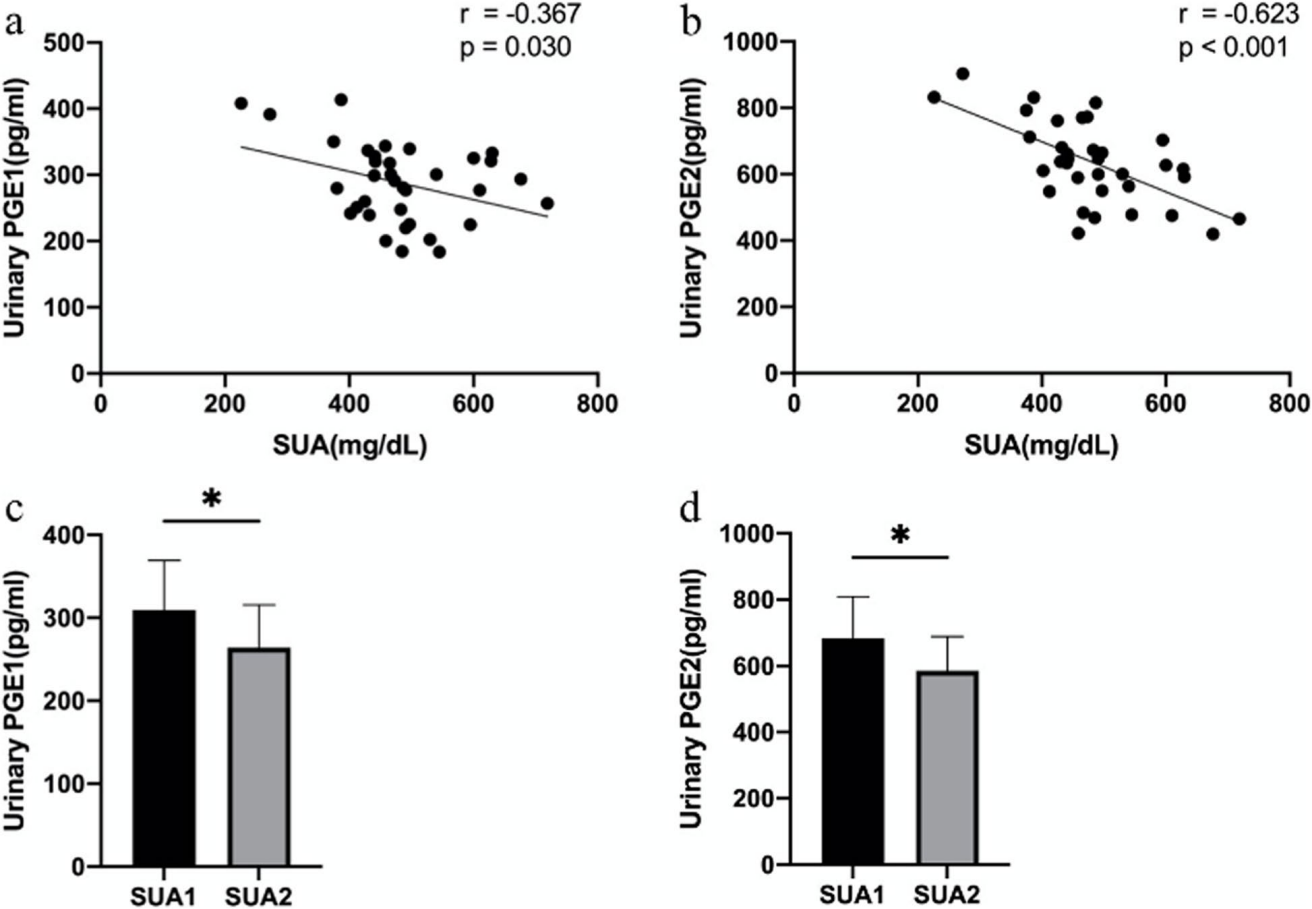
The correlation analysis of SUA and urinary PGs. a Pearson correlation analysis of SUA and urinary PGE1. b Pearson correlation analysis of SUA and urinary PGE2. c Urinary PGE1 levels in different SUA group. d Urinary PGE2 levels in different SUA group. * *P* < 0.05

The results of S-W test show that SUA (*P* = 0.334), Cua (*P* = 0.119), PGE1 (*P* = 0.630), PGE2 (*P* = 0.533), PGI2 (*P* = 0.175), TXA2 (*P* = 0.445) and 6-keto-PGF1a (*P* = 0.054) follow the normal distribution, but FEua (*P* = 0.010), PGD (*P* = 0.007) and TXB2 (*P* = 0.029) do not follow the normal distribution. To detect the correlation of SUA and urinary prostaglandins, we analyzed the correlation between SUA and urinary prostaglandins. We found that SUA was negatively correlated with urinary PGE1(*r* = -0.367, *P* = 0.030) and PGE2(*r* = -0.623, *P* < 0.001) (Fig. 1a, b). However, there was no significant correlation between SUA and other urinary PGs. After adjusting the covariates (Age, BMI, eGFR, TG, TC, HDL and LDL), SUA was still negatively correlated with PGE1 (*r* = -0.615, *P* = 0.009) and PGE2 (*r* = -0.824, *P* < 0.001) (Table 1). Then, Patients were divided into SUA1 group (SUA < 482.6 mg/dL) and SUA2 group (SUA $\ge$ 482.6 mg/dL). Compared with SUA1, both urinary PGE1 (*P* = 0.022) and PGE2 (*P* = 0.019) were lower in SUA2 group (Fig. 1c, d). These results were consistent with the results of correlation analysis.

**Table 1:** Correlation analysis between SUA and urinary prostaglandins after adjusting the covariates

	SUA	*P* value
PGD	-0.356	0.160
PGE1	-0.615	0.009**
PGE2	-0.824	< 0.001***
PGI2	0.278	0.280
TXA2	-0.450	0.070
TXB2	0.273	0.345
6-keto-PGF1a	0.434	0.121

^*^ *P* < 0.05, ** *P* < 0.01, *** *P* < 0.001

### The correlation between urinary prostaglandins and urinary uric acid excretion

**Fig. 2:**
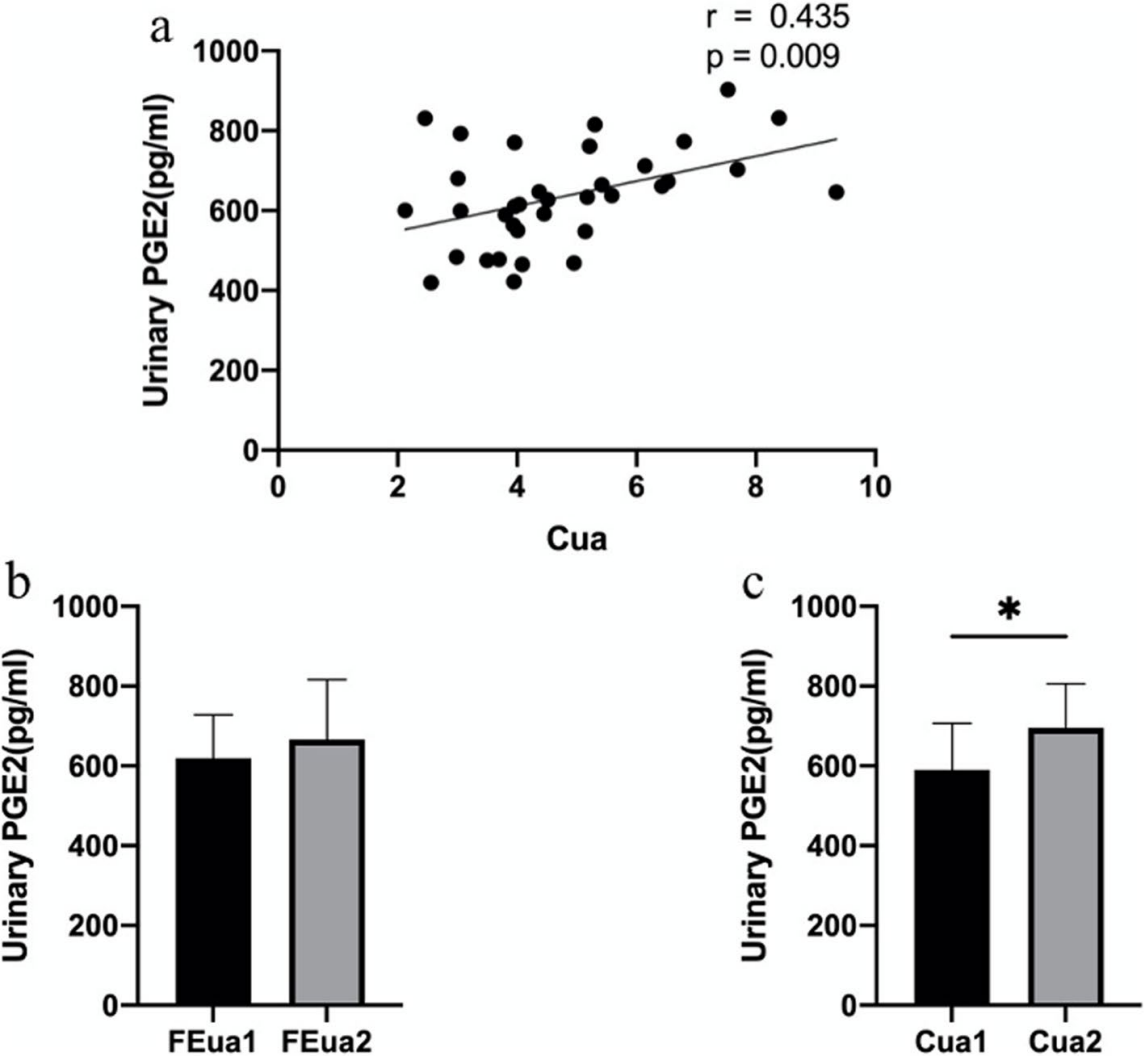
Difference of PGE2 in different FEua and Cua groups. a Pearson correlation analysis of Cua and urinary PGE2. b Urinary PGE2 levels in different FEua groups. c Urinary PGE2 levels in different Cua groups. * *P* < 0.05

We found that Cua was positively correlated with urinary PGE2 (*r* = 0.436, *P* = 0.010) (Fig. 2a). After adjusting the covariates (Age, BMI, eGFR, TG, TC, HDL and LDL), we found that both FEua (*r* = 0.585, *P* = 0.028) and Cua (*r* = 0.637, *P* = 0.014) were positively correlated with urinary PGE2 (Table 2).

**Table 2:** Correlation analysis between urinary acid excretion and urinary prostaglandins after adjusting the covariates

	FEua	*P* value	Cua	*P* value
PGD	0.317	0.215	0.216	0.405
PGE1	0.541	0.025*	0.273	0.288
PGE2	0.556	0.020*	0.518	0.033*
PGI2	-0.209	0.420	-0.221	0.415
TXA2	0.233	0.367	0.370	0.143
TXB2	-0.137	0.641	-0.289	0.317
6-keto-PGF1a	-0.408	0.147	-0.431	0.124

Pearson correlation analysis was used when both groups of data followed normal distribution, otherwise Spearman correlation analysis was used. * *P* < 0.05

The multiple linear regression results clearly indicate that Cua was positively correlated with PGE2 after adjusting for potential confounders (Age, BMI, eGFR, TG, TC, HDL and LDL) (Table 3).

**Table 3:** Multiple linear regression analysis of urinary PGE2 with Cua

	**B**	**SE**	**Beta**	**t**	***P* value**
Cua	**38.045**	**16.205**	**0.542**	**2.348**	0.033*
Age	**-4.032**	**1.663**	**-0.442**	**-2.424**	**0.028**
BMI	**-5.342**	**7.690**	**-0.224**	**-0.695**	**0.498**
eGFR	**1.093**	**2.019**	**0.108**	**0.541**	**0.596**
TG	**-35.074**	**34.665**	**-0.416**	**-1.012**	**0.328**
TC	**31.959**	**110.457**	**0.224**	**0.289**	**0.776**
HDL	**-211.519**	**156.206**	**-0.385**	**-1.354**	**0.196**
LDL	**7.741**	**121.349**	**0.045**	**0.064**	**0.950**

dependent variable: urinary PGE2

^*^ *P* < 0.05

### Differences of PGE2 in urinary uric acid excretion markers

In order to further study the difference of urinary PGE2 in different groups, we divided patients into two groups using the mean values of FEua and Cua respectively. Patients were divided into FEua1 group (FEua < 4.212%) and FEua2 group (FEua $\ge$ 4.212%) by the mean value of FEua (Table 4). Compared with the FEua1 group, levels of urinary PGE2 were higher in the FEua2 group (Fig. 2b), which was consistent with the results of the correlation analysis. However, no statistical significance was found between the two, which may be related to our small sample size.

**Table 4:** Characteristics of the participants according to the mean value of FEua

Variables	All (*n* = 38)	FEua1 (*n* = 24) FEua < 4.212%	FEua2 (*n* = 14) FEua ≥ 4.212%	*P* value
Age(years)	46.08 ± 13.97	46.96 $\pm$ 12.70	44.57 ± 16.32	0.618
BMI (kg/m^2^)	24.68 ± 4.67	24.36 $\pm 2.32$	25.25 ± 7.21	0.578
eGFR (ml/min/1.73 m^2^)	89.25 ± 13.46	92.36 $\pm 13.88$	85.35 ± 12.37	0.183
SUA	479.71 ± 100.40	513.54 ± 91.39	421.71 ± 90.42	0.005**
BG	5.14 ± 0.70	5.20 ± 0.57	5.04 ± 0.87	0.549
TG	2.35 ± 1.69	2.49 ± 1.52	2.10 ± 2.00	0.583
TC	4.86 ± 0.97	4.92 ± 1.00	4.77 ± 0.97	0.688
24-hUV(mL)	2203.95 ± 951.39	2448.13 $\pm 969.31$	1785.36 ± 784.27	0.036*
24-hUua (mmol)	3.28 ± 1.11	3.26 $\pm 1.10$	3.32 ± 1.16	0.871
PGD (pg/ml)	272.52 ± 175.24	264.72 $\pm 179.99$	287.47 ± 172.50	0.672
PGE1 (pg/ml)	287.56 ± 59.47	283.73 $\pm 52.29$	294.90 ± 73.31	0.605
PGE2 (pg/ml)	635.61 ± 124.21	619.83 ± 108.31	665.87 ± 150.68	0.305
PGI2(pg/ml)	104.86 ± 34.94	103.54 ± 36.10	107.39 ± 34.02	0.762
TXA2(pg/ml)	80.87 ± 34.98	83.31 ± 32.45	80.47 ± 31.41	0.805
TXB2 (pg/ml)	254.62 ± 60.08	268.25 $\pm 64.22$	232.61 ± 46.92	0.093
6-keto-PGF1α (pg/ml)	614.73 ± 228.51	643.02 ± 232.79	569.02 ± 222.73	0.367

^*^ *P* < 0.05, ** *P* < 0.01

Similarly, Patients were divided into Cua1 group (Cua $<$ 4.859 ml/min/1.73) and Cua2 group (Cua $\ge$ 4.859 ml/min/1.73) by the mean value of Cua (Table 5). In Cua2 group, an increase of urinary PGE2(*P* = 0.011) was observed (Fig. 2c).

**Table 5:** Characteristics of the participants according to the mean value of Cua

Variables	All (*n* = 38)	Cua1 (*n* = 20) FEua < 4.859 ml/min/1.73m^2^	Cua2 (*n* = 18) FEua ≥ 4.859 ml/min/1.73m^2^	*P* value
Age(years)	46.08 ± 13.97	46.00 $\pm 14.56$	46.17 ± 13.70	0.971
BMI (kg/m^2^)	24.68 ± 4.67	23.69 $\pm 2.67$	25.78 ± 6.09	0.172
eGFR (ml/min/1.73 m^2^)	89.25 ± 13.46	91.00 $\pm 15.71$	87.35 ± 10.83	0.492
SUA	479.71 ± 100.40	520.10 ± 97.15	434.83 ± 85.66	0.007**
BG	5.14 ± 0.70	5.14 ± 0.60	5.12 ± 0.84	0.936
TG	2.35 ± 1.69	2.39 ± 1.60	2.28 ± 1.88	0.853
TC	4.86 ± 0.97	4.90 ± 0.97	4.81 ± 1.01	0.799
24-hUV(mL)	2203.95 ± 951.39	2071.50 $\pm 874.17$	2351.11 ± 1035.55	0.373
24-hUua (mmol)	3.28 ± 1.11	2.70 $\pm 0.82$	3.92 ± 1.06	*P* < 0.001***
PGD (pg/ml)	272.52 ± 175.24	249.76 $\pm 171.46$	301.46 ± 179.72	0.393
PGE1 (pg/ml)	287.56 ± 59.47	281.11 $\pm 59.73$	296.16 ± 60.07	0.467
PGE2 (pg/ml)	635.61 ± 124.21	590.65 ± 116.51	695.56 ± 110.94	0.011*
PGI2 (pg/ml)	104.86 ± 34.94	107.32 ± 39.90	101.58 ± 28.00	0.638
TXA2(pg/ml)	80.87 ± 34.98	82.52 ± 33.19	82.09 ± 30.65	0.969
TXB2 (pg/ml)	254.62 ± 60.08	269.76 $\pm 65.81$	237.59 ± 49.47	0.121
6-keto-PGF1α (pg/ml)	614.73 ± 228.51	616.55 ± 260.62	612.68 ± 194.65	0.962

^*^ *P* < 0.05, ** *P* < 0.01, *** *P* < 0.001

## Discussion

In our study, we found that SUA was negatively correlated with urinary PGE2, and Cua was positively associated with urinary PGE2.PGE2 was significantly different between different SUA and Cua groups.

Prostaglandin E2(PGE2) is the main product catalyzed synthesis of arachidonic acid through the cycloxidase metabolic pathway, and is highly synthesized in the kidney [16]. Initially, it was widely believed that renal water transport was regulated solely by the hypothalamic production of arginine vasopressin (AVP), also known as antidiuretic hormone [17, 18]. However, abundant evidence suggests that PGE2 also plays an important role in regulating renal collecting tube substance reabsorption. Studies in humans and rodents have found that Cox-2 expression is significantly increased in the dense macula and the thick ascending ramus of cortex when effective blood volume decreases, and its main inducible substances such as PGE2 act on the glomeruli to counter the vasoconstriction effect of endogenous vasoactive substances [19]. In addition to improving renal microcirculation, PGE2 synthesized in the dense plaques can also increase renin release [20]. The release of renin caused by PGE2 mainly occurs in isolated paraglomerular organ (JAG) cells, and its action may be related to the cAMP signal transduction pathway mediated by EP2 or EP4 receptor, but the specific molecular mechanism needs to be further studied. It has been found in animal experiments that EP1 receptor can regulate aquaporin and sodium transporter in kidney, weaken AVP-induced water transport and inhibit sodium transport in mouse collecting tubes, which is mediated by ENaC and Pendrin pathways [21]. A previous study has showed that the kidney PGE2 level is higher in hyperuricemia rat [22].Some other studies have found that high uric acid level can cause the thickening of the afferent arterioles, or impairs the autoregulatory response of the afferent arterioles, producing renal hypoperfusion [23, 24], this sequence of reactions may lead to the activation of RAAS, which in turn leads to the elevation of PGE2.Incresed PGE2 can increase the Tubuloglomerular feedback(TGF) sensitivity [25]. Activation of tubule feedback leads to further elevation of uric acid levels. Urine sodium excretion is positively correlated with urine uric acid excretion [26], which is consistent with our previous studies [27], and FEua is positively correlated with FENa. Therefore, PGE2 may also affect the transport of uric acid in the kidney, and the specific mechanism needs to be further elucidated. In addition to PGE2, other prostaglandins are present in our body. TXA2 and PGI2 are unstable and quickly degrade to TXB2 and 6-keto-PGFLA in aqueous solution. TXA2(TXB2) and PGl2(6-keto-PGF1a) form a pair of biological activities, but are mutually restricted, and are important substances to maintain the stability of the internal environment [28]. The stable metabolites TXB2 and 6-keto-PGF1A could reflect the ratio of TXA2 to PGI2. TXA2 is a vasoconstrictor [29, 30], which plays a key role in the regulation of renal hemodynamics, determined by the use of TP agonists (U-46,619) [31, 32]. A previous study reported that Angii-induced elevated levels of vasoceramide may be involved in the pathogenesis of renal injury through TXA2-mediated vasoconstriction [33, 34]. Another study supported this conclusion, suggesting that the AngII/AT1 receptor /nSMase/ ceramide—PLA2 /TXA2 pathway contributes to the regulation of renal vasoconstriction [35]. It is possible that the amount of TXA2, a vasoconstrictor, decreases and the amount of its metabolite TXB2 in urine decreases. However, further studies are needed to determine the extent to which FEua physiologically regulates the excretion of TXB2 during vascular motility.

Uric acid is mainly excreted in the kidney [36]. Some in vitro studies have found that PGE2 release of cells increases under the stimulation of uric acid [37, 38]. However, these experiments were not confirmed in vivo. After analyzing the urine of patients with hyperuricemia, we found that SUA was significantly negatively correlated with urinary PGE2, while Cua was positively correlated with urinary PGE2. When uric acid excretion increases, uric acid in renal tubules increases, which indicates that uric acid may promote PGE2 synthesis in renal tubular epithelial cells. These results are consistent with the results of previous study [11]. In individuals with the higher FEua and Cua may be accompanied by volume expansion. This can be briefly illustrated by a previous study [39], in which 19 subjects were performed with rapid infusion of hypo-, iso- or hypertonic saline and assessed the relative contributions of volume expansion and increased FENa to the uricosuria of saline infusion. As mentioned above, FEua is positively correlated with FENa, the relationship between FEua and FENa was entirely attributed to their correlation with infusion volume. In other words, individuals with higher FEua and Cua may have imperceptible volume expansion accompanied by less vasoconstriction compared to the lower FEua and Cua.

As far as we know, little results were found in the study on the question of the urinary excretion of prostaglandins based on the renal handling of uric acid. The importance and originality of this study are that it explores that there is an association between uric acid and urinary prostaglandins. Apparently, the present study naturally includes some limitations. A major source of limitation is due to small sample size, which may give rise to considerable deviations. In addition, normal SUA people and women are not included in this study. Finally, the mechanism of the urinary prostaglandins and urinary uric acid is not further studied.

## Conclusions

Our findings provide evidence that SUA was negatively correlated with urinary PGE2, and Cua was positively correlated with urinary PGE2. Urinary PGE2 was significantly different between different SUA and Cua groups. It is suggested that uric acid may be correlated with urinary prostaglandin excretion in patients with hyperuricemia, which further suggests one of the possible causes of abnormal renal hemodynamics in hyperuricemia. Here we bring together much of this work, which has so far only scratched the surface of this very fertile field of the interaction between uric acid and prostaglandins. Detecting the uric acid may has a potential prompting effect on predicting the renal hemodynamic.

## Data Availability

The datasets used and analysed during the current study available from the corresponding author on reasonable request.

## Abbreviations

SUA: Serum uric acid
PGD: Prostaglandin D
PGE1: Prostaglandin E1
PGE2: Prostaglandin E2
TXA2: Thromboxane A2
TXB2: Thromboxane B2
FEua: Fraction of uric acid excretion
Cua: Uric acid clearance rate
UA: Uric acid
CKD: Chronic kidney disease
HUA: Hyperuricemia
PGs: Prostaglandins
Scr: Serum creatinine
BUN: Blood urinary nitrogen
TC: Total cholesterol
TG: Total triglycerides
HDL: High-density lipoprotein
LDL: Low-density lipoprotein
FBG: Fasting blood glucose
eGFR: Estimated glomerular filtration rate
AVP: Arginine vasopressin
JAG: Isolated paraglomerular organ
TGF: Tubuloglomerular feedback
